# miR-6315 Attenuates Methotrexate Treatment-Induced Decreased Osteogenesis and Increased Adipogenesis Potentially through Modulating TGF-β/Smad2 Signalling

**DOI:** 10.3390/biomedicines9121926

**Published:** 2021-12-16

**Authors:** Ya-Li Zhang, Liang Liu, Yu-Wen Su, Cory J. Xian

**Affiliations:** UniSA Clinical and Health Sciences, University of South Australia, Adelaide, SA 5000, Australia; yali.zhang@mymail.unisa.edu.au (Y.-L.Z.); liang.liu@csl.com.au (L.L.); yu-wen.su@unisa.edu.au (Y.-W.S.)

**Keywords:** methotrexate, miR-6315, bone formation, marrow adiposity, TGF-β, Smad2

## Abstract

Methotrexate (MTX) treatment for childhood malignancies has shown decreased osteogenesis and increased adipogenesis in bone marrow stromal cells (BMSCs), leading to bone loss and bone marrow adiposity, for which the molecular mechanisms are not fully understood. Currently, microRNAs (miRNAs) are emerging as vital mediators involved in bone/bone marrow fat homeostasis and our previous studies have demonstrated that miR-6315 was upregulated in bones of MTX-treated rats, which might be associated with bone/fat imbalance by directly targeting Smad2. However, the underlying mechanisms by which miR-6315 regulates osteogenic and adipogenic differentiation require more investigations. Herein, we further explored and elucidated the regulatory roles of miR-6315 in osteogenesis and adipogenesis using in vitro cell models. We found that miR-6315 promotes osteogenic differentiation and it alleviates MTX-induced increased adipogenesis. Furthermore, our results suggest that the involvement of miR-6315 in osteogenesis/adipogenesis regulation might be partially through modulating the TGF-β/Smad2 signalling pathway. Our findings indicated that miR-6315 may be important in regulating osteogenesis and adipogenesis and might be a therapeutic target for preventing/attenuating MTX treatment-associated bone loss and marrow adiposity.

## 1. Introduction

Bone marrow stromal cells (BMSCs) have the potential to undergo multiple differentiation into diverse cell types, such as osteoblasts, adipocytes, and chondrocytes [1,2]. Commitment and differentiation into various lineages are tightly regulated by the extracellular environment, and changes in the signals and/or molecules can result in abnormal differentiation of BMSCs [3]. Chemotherapy is the most vital modality for treating childhood cancers, owing to a cure rate of higher than 75% [4]. However, lifelong bone-related side effects from chemotherapy have increased, such as bone growth impairments, low bone mass, marrow adiposity, and increased fracture risks, thus decreasing patient life quality [4]. With the greater success in treating cancers, the focus now has turned to understanding how chemotherapy damages bone and minimizing these bone defects.

Antimetabolite methotrexate (MTX) is known as a cancer chemotherapeutic agent and immune system suppressant, which has been applied to treat autoimmune diseases and cancers [5,6,7,8]. Recently, Krahel et al. found that proprotein convertase subtilisin/kexin type 9 (PCSK9) was associated with lipid disturbances and could be a novel biomarker in psoriasis, and that three months of MTX treatment remarkably reduced the PCSK9 levels in psoriasis patients, indicating that MTX might be considered as a new treatment approach for psoriasis [9]. Although MTX has shown high pharmacological efficacy, MTX-associated side effects in clinical applications are not negligible. A systematic review summarized the adverse effects of MTX in ophthalmology and it was found that, despite being considered well-tolerated and reversible, even the low-dose of MTX causes significant toxicity, such as central nervous system complications, mucositis, and hepatotoxicity, with substantial death rates [10]. Furthermore, MTX treatment has shown effects on blastocyst formation, resulting in dysmorphic features and neurologic defects during early pregnancy [10]. Furthermore, MTX treatment during childhood might lead to visual defects in patients [10]. Additionally, various clinical studies have demonstrated that intensive MTX treatment causes skeletal growth defects, such as reduced bone mass and increased marrow fat contents [11,12]. These clinical observations were also supported by animal studies in rats in which the increased adipogenic differentiation potential and the declined osteogenic differentiation potential were found in the bone marrow following MTX treatment [13,14]. It was indicated that MTX treatment suppressed the expression of osteogenesis-related genes, such as runt-related transcription factor 2 (RUNX2) and osterix (OSX), disrupting osteoblast differentiation and function [13,14]. On the other hand, previous in vivo and in vitro investigations illustrated that MTX chemotherapy resulted in marrow adiposity via inducing the expression of adipogenesis-related genes (e.g., peroxisome proliferator activated receptor gamma (PPARγ)) [13,14].

In order to prevent/reduce MTX-induced side effects, the supplementation of folate (folic acid or folinic acid) is common among patients treated with low-dose MTX [10]. Fan et al. also observed that a folinic acid supplementation preserves the formation of the primary spongy bone and bone growth during long-term low-dose MTX therapy in rats [15]. On the other hand, although folate supplementation decreases the MTX-associated hepatotoxicity and gastrointestinal in patients with rheumatoid arthritis (RA), no statistically significant differences were identified between high and low doses of folate treatment on MTX efficacy or toxicity [16].

However, therapeutic approaches for MTX-induced bone loss and marrow adiposity are currently lacking and the associated molecular mechanisms are not fully understood. MicroRNAs (miRNAs) are non-coding RNA molecules that regulate mRNA silencing by interacting with the 3′ untranslated region (3′ UTR) of targets [17,18]. Currently, miRNAs are emerging as essential mediators involved in regulating BMSC differentiation [3,19,20,21]. Previously, the regulation of the miRNA-6315 network was found to be potentially involved in hyperplasia of mammary glands and cell proliferation during liver regeneration [22,23]. Our recent study has shown that miR-6315 was upregulated in bones of MTX-treated rats, which might be associated with bone/bone marrow fat imbalance by directly targeting Smad2 [24]. However, the underlying mechanisms by which miR-6315 regulates osteogenic and adipogenic differentiation remain unknown.

Herein, we investigated the regulatory roles of miR-6315 in osteogenesis and adipogenesis using in vitro cell culture model systems with MC3T3.E1 preosteoblastic cells and 3T3 F442A preadipocytic cells, respectively. These cell lines have been widely used as in vitro cell models for osteogenesis and adipogenesis due to their differentiation-inducing capacity [25,26,27]. We found that miR-6315 exerts a greater impact on preadipocyte viability and apoptosis than preosteoblasts. miR-6315 also promotes osteogenic differentiation and attenuates MTX-induced increased adipogenesis. Furthermore, our results suggested that the action of miR-6315 involved in osteogenesis/adipogenesis regulation might be partially through modulating the TGF-β/Smad2 signalling pathway.

## 2. Materials and Methods

### 2.1. Cell Culture and In Vitro Osteoblastic Differentiation

MC3T3.E1 cells (CellBank Australia, Westmead, NSW, Australia) were seeded in 6-well/12-well plates with basal medium containing α-MEM (Sigma-Aldrich, North Ryde, NSW, Australia), 15 mM HEPES (Thermo Fisher Scientific, Scoresby, VIC, Australia), 10% FBS (Invitrogen, Carlsbad, CA), 2 mM L-glutamine (Sigma), and 1% antibiotic-antimycotic (Thermo Fisher Scientific) until 70–80% confluence [28]. To induce osteogenesis of the confluent cells, the basal medium was supplemented with 10 mM β-glycerol-2-phosphate and 10 nM dexamethasone (Sigma) (Sigma). Some cells were also treated with/without 10 μM of MTX for 48 h [29], followed by transfection of the miR-6315 agomir or negative control (BioNovus Life Sciences, Cherrybrook, NSW, Australia) with Lipofectamine^®^ 2000 reagent (Thermo Fisher Scientific). The treated cells were cultured in a medium that was refreshed every 2 days until further experiments.

### 2.2. Alizarin Red S (ARS) Staining for Mineralization

At Days 21–23, osteogenically differentiated cells were stained by 40 mM Alizarin Red S (ARS, Sigma) after being fixed by 10% formalin (Sigma), as described [28]. After being washed with water and observed and photographed using a microscope, the ARS stain was solubilized by 10% acetic acid, and absorbance was read at 405 nm, as described [28].

### 2.3. Cell Culture and In Vitro Adipocytic Differentiation

3T3 F442A cells (CellBank Australia) were seeded in 6-well/12-well plates with culture medium containing DMEM (Sigma), 10% newborn calf serum (NBCS, Sigma) and 2 mM L-glutamine until 70–80% confluence [28]. To induce adipogenesis of the confluent cells, the culture medium was supplemented with 5 mg/mL insulin (Sigma). Some cells were treated with/without 10 μM of MTX for 48 h, followed by transfection of the miR-6315 agomir or negative control using Lipofectamine^®^ 2000 reagent. The treated cells were cultured with medium being refreshed every 2 days until further experiments.

### 2.4. Oil Red O (ORO) Staining for Adipogenesis

At Days 14–17, after being fixed with 10% formalin, adipogenically differentiated cells were incubated with 60% isopropyl alcohol (Sigma) for 5 min, and then stained with Oil Red O (ORO, Sigma) for 30 min. After washes with diH_2_O, cells were stained with hematoxylin (Sigma) for 1 min prior to being photographed using a microscope. Then the ORO stain was extracted with 1 mL of 100% isopropyl alcohol (5 min with gentle rocking) and absorbance was read (in triplicate) at 490 nm.

### 2.5. Cell Viability Assay (MTT Assay)

MC3T3.E1 cells/3T3 F442A cells were seeded into 96-well plates with basal medium until 70–80% confluence. To examine the treatment effects, cells were treated with/without 10 μM of MTX for 48 h, followed by transfection with the miR-6315 agomir or negative control as described above. After 48 h, cells were treated with a medium containing 0.5 mg/mL MTT (Sigma) and then incubated for further 2 h. The MTT-containing medium was removed and replaced with 100 µL DMSO (Sigma). Plates stood at room temperature for 20 min and were gently shaken before absorbance was read at 595 nm.

### 2.6. Cell Apoptosis Assay

MC3T3.E1 cells/3T3 F442A cells were seeded in 96-well plates with basal medium until 70–80% confluence. To examine treatment effects, cells were treated with/without 10 μM of MTX for 48 h, followed by transfection with the miR-6315 agomir or negative control (48 h). Then the treatment-containing medium was replaced with 4 µM (~100 µL/per well) of CellEvent™ Caspase-3/7 green detection reagent (Thermo Fisher Scientific) and incubated at 37 ℃ for 30 min. Cells were fixed with 10% formalin (Sigma) for 15 min and then were stained with 2 µg/mL DAPI (Sigma) in the dark for 5 min. Stained cells were imaged using ZOE™ fluorescent cell imager (Bio-Rad Laboratories, Gladesville, NSW, Australia).

### 2.7. Signalling Pathway Analyses

MC3T3.E1 cells/3T3 F442A cells were seeded in 6-well plates until 70–80% confluence and then cells were induced for osteogenic or adipogenic differentiation, as described above. After cells were transfected with the miR-6315 agomir or negative control for 48 h, as described above, the differentiation medium was supplemented with/without 1 ng/mL of TGF-β1 (Lonza, Brooklyn, VIC, Australia). Treatment effects on expression of the TGF-β/Smad2 signalling pathway target genes were analysed by RT-qPCR and Western blot, as described below.

### 2.8. RNA Isolation and RT-qPCR

Total RNA from cells was isolated using a GenElute™ Total RNA Purification Kit (Sigma). First, strand cDNA was reverse transcribed using iScript™ Advanced cDNA Synthesis Kit (Bio-Rad). Specific primers (Table 1, designed using NCBI Primer Blast and Primer 3.0 InPut software) were synthesized by Sigma-Aldrich. Using the cDNA samples, quantitative PCR assays (in triplicate) were performed using SsoAdvanced™ Universal SYBR^®^ Green Supermix (Bio-Rad) and a CFX PCR system (Bio-Rad). After being normalized to the expression of internal housekeeping gene cyclophilin A (CycA) [13,28,30,31], the relative expression of genes of interest was calculated with the comparative 2^−ΔΔCt^ method.

### 2.9. Western Blot

Cellular protein was extracted using RIPA lysis buffer (Thermo Fisher Scientific) containing 10 µL/mL protease and phosphatase inhibitors (Thermo Fisher Scientific), as described [28]. A protein assay was performed using a BCA assay kit (Thermo Fisher Scientific) to measure the protein concentrations in samples. Then protein samples (at 8 µg) were mixed with Laemmli buffer (Bio-Rad) and loaded onto 4–20% SDS-PAGE gels (Bio-Rad). Chameleon^®^ Duo Pre-stained protein ladder (LI-COR, Mulgrave, VIC, Australia) was used as molecular weight markers. Electrophoresis was conducted at a constant voltage of 90 V for 10 min and then 120 V for about 40 min with Novex™ Tris-Glycine SDS running buffer (Thermo Fisher Scientific). Proteins were then transferred to 0.2 µm nitrocellulose transfer packs (Bio-Rad) using Trans-Blot Turbo Transfer System (Bio-Rad). After the protein transfer, membranes were stained with Revert™ 700 total protein stain (LI-COR). Then membranes were rinsed with Revert™ 700 wash solution, followed by imaging and quantification in the 700 nm channel with an Odyssey^®^ CLx imaging system (LI-COR). After being blocked with 3% milk/TBST blocking buffer at room temperature for 1 h, membranes were incubated overnight at 4 ℃ with a specific primary antibody (anti-Smad2 antibody (ab33875, Abcam, VIC, Australia) or anti-DLX5 polyclonal antibody (PA5-101134, Thermo Fisher Scientific)). The blots were then rinsed with 1 X TBST before being incubated in an IRDye^®^ 800 CW donkey anti-rabbit IgG secondary antibody (LI-COR) solution for 1 h at room temperature. After being washed with 1 X TBST, the immunodetection was visualized in the 800 nm channel with an Odyssey^®^ CLx imaging system (LI-COR). Protein expression levels were quantified using Image Studio Lite Ver 5.2 software (LI-COR).

### 2.10. Statistical Analyses

Data were analysed by GraphPad Prism 8 (GraphPad Software, Inc., San Diego, CA, USA). A Shapiro–Wilk test with QQ plot was performed for testing data normality and lognormality prior to statistical analyses. Statistical significance of the differences between groups (*p* < 0.05 being considered as statistically significant) was performed via *t*-tests or one-way ANOVA followed by Tukey’s post-test, as mentioned in each figure legend. Statistical significance (comparing between each treatment and the negative control) was marked with * *p* < 0.05, ** *p* < 0.01, *** *p* < 0.001, or **** *p* < 0.0005. Experiments were performed at least three times (*n* = 3) and the results are displayed as the mean ± SEM.

## 3. Results

### 3.1. miR-6315 Treatment Effects on Preosteoblast Viability and Apoptosis

**Figure 1:**
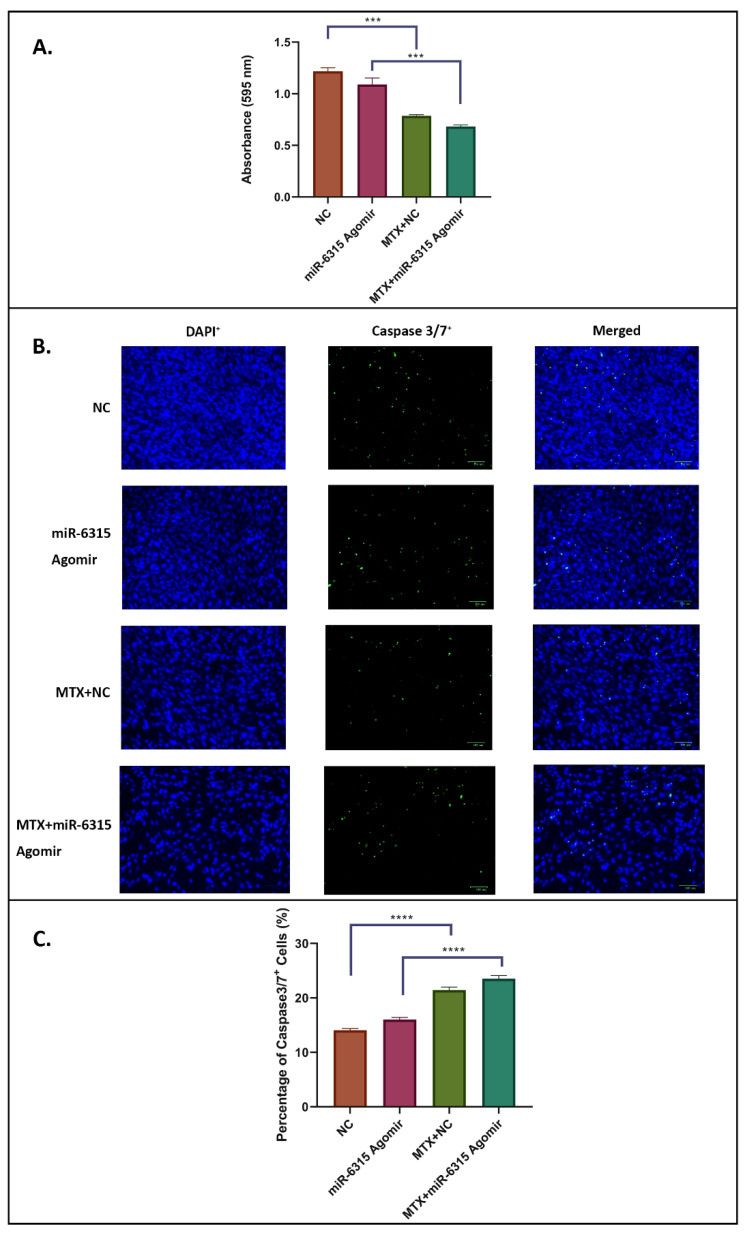
Effects of miR-6315 on osteoblastic cell viability and apoptosis. MC3T3.E1 cells were cultured until 80% confluence and treated with negative control (NC) or miR-6315 agomir alone, with methotrexate (10 μM MTX)+NC or with MTX (10 μM)+miR-6315 agomir for 48 h, and then cell viability and apoptosis were examined by MTT assay and apoptosis assay, respectively. (**A**) Comparisons of cell viability as measured by MTT assay. (**B**) Representative images of DAPI-labelled (blue colour) cells and caspase3/7 immunofluorescent-labelled (green colour) apoptotic cells in the treated groups. Scale bar on panel B = 100 µm. (**C**) Total caspase 3/7^+^ cells were quantified and expressed as a percentage of the total DAPI^+^ cells. Statistical significance was marked with *** *p* < 0.001, or **** *p* < 0.0005. Experiments were performed at least three times and the results are displayed as the mean ± SEM (*n* = 3). Statistical significance was performed via one-way ANOVA, followed by Tukey’s post-test.

As a means to explore the possibility that miR-6315 could affect MC3T3.E1 preosteoblast viability and apoptosis, effects of MTX treatment and/or transfection with miRNA-6315 or the NC control on viability and apoptosis were examined in preosteoblasts. Although a slight decrease in preosteoblast viability (examined by the MTT assay) was observed in the miR-6315 agomir-treated group, no significant differences were identified between the miR-6315-treated group and the NC-treated group (*p* = 0.0733) (Figure 1A). A similar pattern was also observed in the comparison between the MTX+miR-6315- and MTX+NC-treated groups (*p* = 0.0513) (Figure 1A). On the other hand, when compared to the NC group, a significantly lower cell viability was observed in the MTX+NC-treated group (*p* < 0.001, Figure 1A). Similarly, there was a reduction in the MC3T3.E1 cell viability in the MTX+miR-6315 agomir combination treatment group when compared with the miR-6315 agomir-treated group (*p* < 0.001, Figure 1A).

Caspase 3/7 immunofluorescent labelling of MC3T3.E1 cells was conducted in an apoptosis assay (Figure 1B,C). Total caspase 3/7^+^ cells (green colour) were quantified and expressed as a percentage of total DAPI^+^ cells (Figure 1B). While considerable differences in the densities of caspase 3/7^+^ cells were observed in the MTX-treated groups (MTX+NC/MTX+miR-6315 agomir) when compared to non-MTX-treated groups (NC/miR-6315 agomir) (Figure 1B,C), consistent with the MTT assay results, there were no obvious changes in the percentages of caspase 3/7^+^ cells observed between the miR-6315 agomir/MTX+miR-6315 agomir groups and NC/MTX+NC groups. Our MTT and apoptosis assay results demonstrated that, while the MTX treatment alone drastically decreased the preosteoblast cell viability and increased cell apoptosis, the miR-6315 agomir treatment had no significant influence on cell viability and apoptosis of these preosteoblastic cells.

### 3.2. miR-6315 Treatment Effects on Preadipocyte Viability and Apoptosis

**Figure 2:**
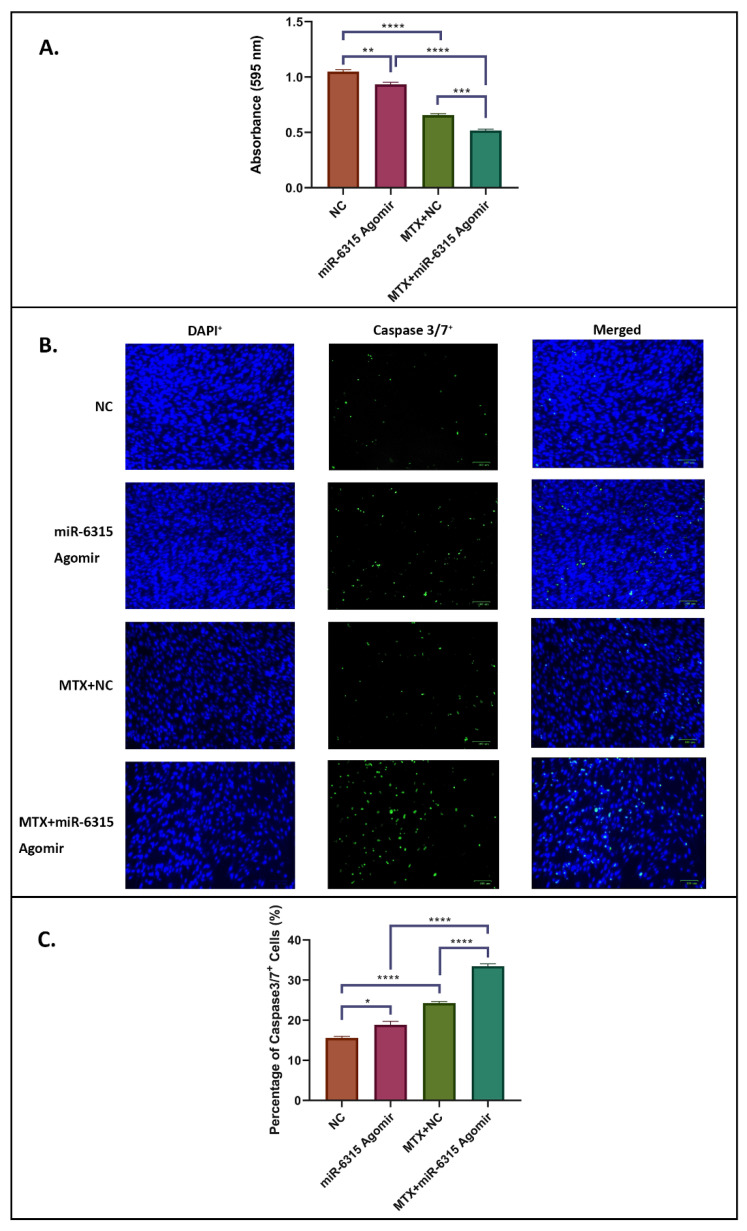
Effects of miR-6315 on preadipocytic cell viability and apoptosis. 3T3 F442A cells were cultured until 80% confluence and treated with the negative control (NC) or miR-6315 agomir alone or with methotrexate (10 μM MTX)+NC or MTX (10 μM)+miR-6315 agomir for 48 h, and then cell viability and apoptosis was examined by MTT assay and apoptosis assay, respectively. (**A**) Comparisons of cell viability as measured by MTT assay. (**B**) Representative images of DAPI labelling (blue colour) cells and caspase3/7 immunofluorescent labelling (green colour) cells in treated groups. Scale bar on panel B = 100 µm. (**C**) Total caspase 3/7^+^ cells were quantified and expressed as a percentage of total DAPI^+^ cells. Statistical significance was marked with * *p* < 0.05, ** *p* < 0.01, *** *p* < 0.001, or **** *p* < 0.0005. Experiments were performed at least three times and the results are displayed as the mean ± SEM (*n* = 3). Statistical significance was performed via one-way ANOVA, followed by Tukey’s post-test.

In order to examine the treatment effects on viability and apoptosis of preadipogenic cells, 3T3 F442A cells were cultured to 80% confluence and treated with NC or miR-6315 agomir alone or with MTX (10 μM)+NC or MTX (10 μM)+miR-6315 agomir for 48 h, and then cells were subjected to MTT assay and apoptosis assay, respectively. A significant decrease in preadipocyte viability was observed in the miR-6315-treated group when compared to the NC group (*p* < 0.01, Figure 2A). Meanwhile, cell viability was remarkably lower in the MTX+miR-6315-treated group than the MTX+NC-treated group (*p* < 0.001, Figure 2A). Furthermore, with MTX treatment (MTX+NC-treated group and MTX+miR-6315 agomir-treated group), considerable declines in cell viability were observed when compared with non-MTX-treated groups (NC-treated group and miR-6315 agomir-treated group) (Figure 2A).

Immunofluorescent caspase 3/7 labelling of 3T3 F442A cells was performed in an apoptosis assay (expressed as a percentage of total DAPI+ cells) (Figure 2B,C). Considerable increases in caspase 3/7^+^ (green colour) cell densities were observed in the MTX-treated groups (MTX+NC/MTX+miR-6315 agomir) when compared to the non-MTX-treated groups (NC/miR-6315 agomir) (*p* < 0.0005, Figure 2B,C). Significant changes also were observed in the percentage of caspase 3/7^+^ cells in the miR-6315 agomir-treated group when compared with the NC group (*p* < 0.05). The same pattern of changes in apoptosis was found in the MTX+miR-6315 group when compared to the MTX+NC group (*p* < 0.0005). These findings illustrated that the MTX treatment and/or miR-6315 supplementation has remarkable impacts on preadipocyte cell viability and apoptosis.

### 3.3. miR-6315 Promotes Osteogenesis and Matrix Mineralization and Attenuates the Inhibitory Effects of MTX Treatment

**Figure 3:**
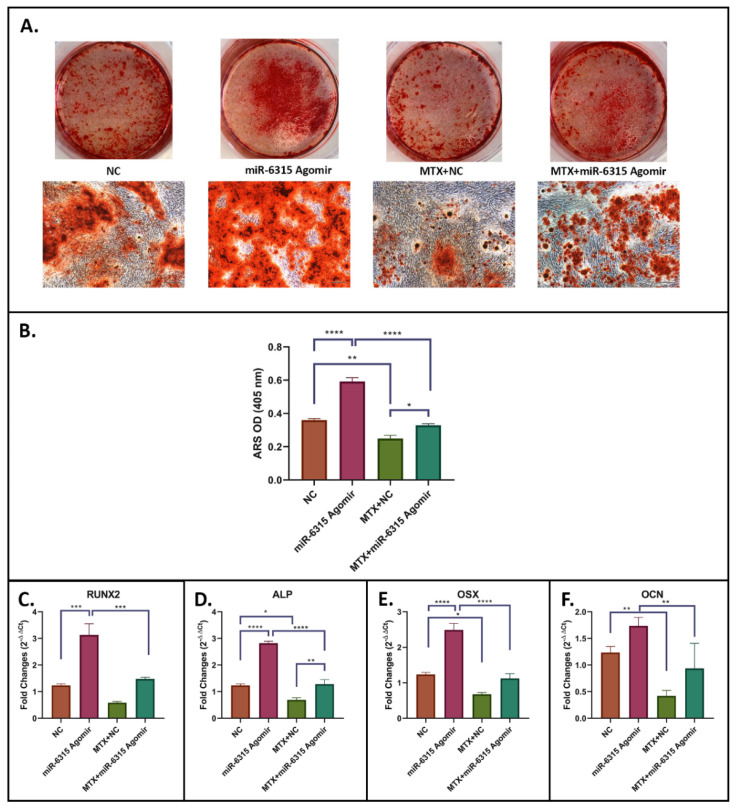
miR-6315 promotes osteogenesis and matrix mineralization and attenuates MTX inhibitory effects. MC3T3.E1 cells were cultured until 80% confluence prior to being used for examining effects on osteogenic differentiation of treatment with the negative control (NC) or miR-6315 agomir alone or with methotrexate (10 μM MTX)+NC or MTX (10 μM)+miR-6315 agomir for 48 h. (**A**) Alizarin Red S (ARS) staining at Day 23 of osteogenic differentiation. Scale bar on panel A = 200 µm. (**B**) ARS quantification. (**C**–**F**) RT-qPCR analyses of osteogenesis-related gene markers at Day 23 of osteogenic differentiation. CycA was used as an internal housekeeping gene. Statistical significance was marked with * *p* < 0.05, ** *p* < 0.01, *** *p* < 0.001, or **** *p* < 0.0005. Experiments were performed at least three times and the results are displayed as the mean ± SEM (*n* = 3). Statistical significance was performed via one-way ANOVA followed by Tukey’s post-test.

To examine the potential effects of miR-6315 on osteogenesis, MC3T3.E1 cells were cultured until 80% confluence prior to being subjected to osteogenic differentiation with or without treatment with NC or miR-6315 agomir alone or with MTX (10 μM)+NC or MTX (10 μM)+miR-6315 agomir for 48 h. At the end of osteogenic differentiation (Day 23), a mineralization assay was used to examine the treatment effects on osteoblast differentiation and matrix mineralization. Compared to the NC alone treatment, there was a significantly higher number of calcified nodules and a higher level of matrix mineralization in the miR-6315-treated group (*p* < 0.0005) (Figure 3A,B). MTX treatment caused a remarkable decrease in the formation of mineralized nodules when compared to NC (*p* < 0.01, NC vs. MTX+NC) (Figure 3A,B). This reduction was attenuated with the MTX+miR-6315 combination treatment (*p* < 0.05, MTX+NC vs. MTX+miR-6315) (Figure 3A,B).

RT-qPCR analyses were performed to assess the treatment effects on the expression of osteogenesis-related transcription factors and markers. A significant upregulation in mRNA expression of RUNX2 (*p* < 0.001), alkaline phosphatase (ALP) (*p* < 0.0005) and OSX (*p* < 0.0005) was observed in the miR-6315-treated group in comparison with the NC group (Figure 3C–E). Compared to the NC group, there was a tendency of increased expression of osteocalcin (OCN) in the miR-6315-treated group (statistically insignificant) (*p* = 0.0777, Figure 3F). The mRNA expression of osteogenesis-related genes in the MTX+NC treatment group shared similar patterns of changes in the numbers of mineralized nodules, with an obvious decline following MTX treatment (NC vs. MTX+NC), which was dampened when the MTX treatment was combined with miR-6315 agomir (MTX+NC vs. MTX+miR-6315, RUNX2, *p* = 0.0537; ALP, *p* < 0.01; OSX, *p* = 0.0869; OCN, *p* = 0.0654, Figure 3C–F). These findings indicated that the MTX treatment inhibited osteogenesis and matrix mineralization while miR-6315 supplementation was able to attenuate MTX-induced defects in osteogenesis.

### 3.4. miR-6315 Inhibits Adipogenesis and Lipid Accumulation and Attenuates the Stimulatory Effects of MTX Treatment

**Figure 4:**
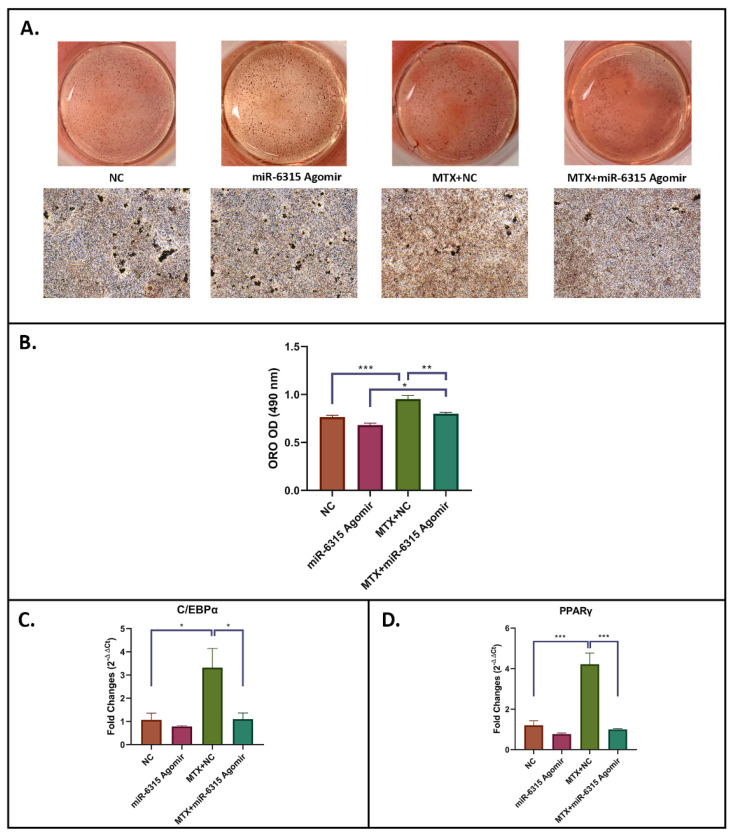
miR-6315 inhibits adipogenesis and lipid accumulation and attenuates MTX stimulatory effects. 3T3 F442A cells were cultured until 80% confluence and then used for adipogenic differentiation with or without treatment with NC or miR-6315 agomir alone or with methotrexate (10 μM MTX)+NC or MTX (10 μM)+miR-6315 agomir for 48 h. (**A**) Oil Red O (ORO) staining was performed at Day 17 of osteogenic differentiation. Scale bar on panel A = 200 µm. (**B**) ORO quantification. (**C**,**D**) RT-qPCR analyses of adipogenesis-related gene markers at Day 17 of adipogenic differentiation. CycA was used as an internal housekeeping gene. Statistical significance was marked with * *p* < 0.05, ** *p* < 0.01, or *** *p* < 0.001. Experiments were performed at least three times and the results are displayed as the mean ± SEM (*n* = 3). Statistical significance was performed via one-way ANOVA followed by Tukey’s post-test.

To examine the potential effects of miR-6315 on adipogenesis, 3T3 F442A cells were cultured until 80% confluence prior to being used for adipogenic differentiation with or without treatment with NC or miR-6315 agomir alone or with MTX (10 μM)+NC or MTX (10 μM)+miR-6315 agomir for 48 h. At the end of adipogenic differentiation (Day 17), an adipogenesis assay was used to examine the treatment effects on adipocyte differentiation and lipid droplet formation. Compared to the NC group, a slight decrease in formation of lipid droplets was found in the miR-6315-treated group (*p* = 0.0960, Figure 4A,B). Furthermore, MTX treatment caused a considerable increase in the production of lipid droplets when compared to the NC group (*p* < 0.001, NC vs. MTX+NC) (Figure 4A,B). On the other hand, treatment with miR-6315 along with MTX attenuated this increased fat formation following MTX treatment (*p* < 0.01, MTX+NC vs. MTX+miR-6315) (Figure 4A,B).

RT-qPCR analyses were performed to assess the treatment effects on the expression of adipogenesis regulatory transcription factors. No significant changes in the levels of C/EBPα mRNA expression were observed in the miR-6315 alone-treated group compared to the NC group (*p* = 0.9699, Figure 4C). The mRNA expression of C/EBPα was significantly upregulated in MTX-treated cells when compared to non-treated controls (*p* < 0.05, NC vs. MTX+NC), while this increase was notably alleviated in MTX-treated group supplemented with miR-6315 (*p* < 0.05, MTX+NC vs. MTX+miR-6315) (Figure 4C). Similarly, mRNA expression of PPARγ was not obviously changed in the miR-6315 alone treatment group (*p* = 0.7468, NC vs. miR-6315), but again dramatically upregulated in the MTX+NC treatment group (*p* < 0.001, NC vs. MTX+NC) (Figure 4D). The MTX treatment along with miR-6315 alleviated this upregulation (*p* < 0.001, MTX+NC vs. MTX+miR-6315) (Figure 4D). These results demonstrated that MTX treatment enhanced adipogenesis and lipid droplet formation and miR-6315 supplementation attenuated MTX-induced adipogenesis.

### 3.5. miR-6315 Represses Smad2 Expression and Downregulates TGF-β Signalling

**Figure 5:**
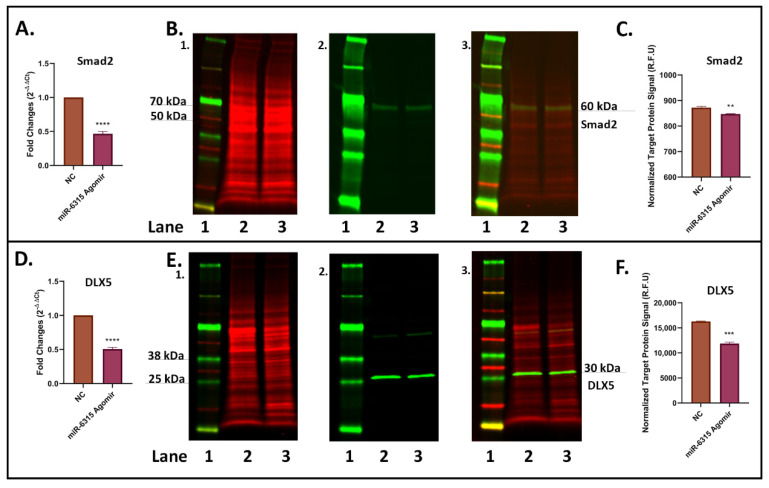
Expression of Smad2 and DLX5 in osteoblastic cells with miRNA-6315 transfection. MC3T3.E1 cells were treated with miR-6315 agomir or the negative control (NC) for 48 h prior to being harvested for RT-qPCR and Western blot analyses. (**A**) RT-qPCR analyses of Smad2. (**B**) Western blot studies of Smad2: (**B**(1)) Total protein extract was visualized (700 nm channel, red). (**B**(2)) Target protein Smad2 was visualized (800 nm channel, green). (**B**(3)) Merged images of blots. (**C**) Normalized target Smad2 protein signals. (**D**) RT-qPCR analyses of distal-less homeobox 5 (DLX5). (**E**) Western blot studies of DLX5. (**E**(1)) Total protein extract was visualized (700 nm channel, red). (**E**(2)) Target protein DLX5 was visualized (800 nm channel, green). (**E**(3)) Merged images of blots. (**F**) Normalized target DLX5 protein signals. Statistical significance was marked with ** *p* < 0.01, *** *p* < 0.001, or **** *p* < 0.0005. Statistical significance analyses were performed via *t*-test. Representative gels of three experiments. Treatments on each lane: lane 1: pre-stained protein ladder; lane 2: transfection with NC; lane 3: transfection with miR-6315 agomir. R.F.U: Relative Fluorescence Units.

To understand the effects of miR-6315 on expression of target gene Smad2 in osteogenic cells, MC3T3.E1 cells were treated with NC or miR-6315 agomir for 48 h prior to being harvested for RT-qPCR and Western blot analyses. Expression of Smad2 in miR-6315-overexpressed MC3T3.E1 cells was found to be markedly declined at both the mRNA and protein levels (Figure 5A–C), suggesting that miR-6315 regulates Smad2 expression at both transcriptional and post-transcriptional levels in osteoblastic cells.

To examine if miR-6315 treatment has impact on TGF-β signalling in osteoblastic cells, the mRNA and protein expression of the signalling downstream gene distal-less homeobox 5 (DLX5) was also measured. It was found that the effects of miR-6315 treatment on DLX5 expression shared similar patterns of changes in the mRNA and protein expression as in Smad2, with notable declines upon miR-6315 treatment (Figure 5D–F), indicating that miR-6315 downregulated Smad2 expression in osteoblastic cells, which may result in suppression of TGF-β signalling.

### 3.6. miR-6315 Regulates Osteogenesis Partially through Affecting TGF-β/Smad2 Signalling

**Figure 6:**
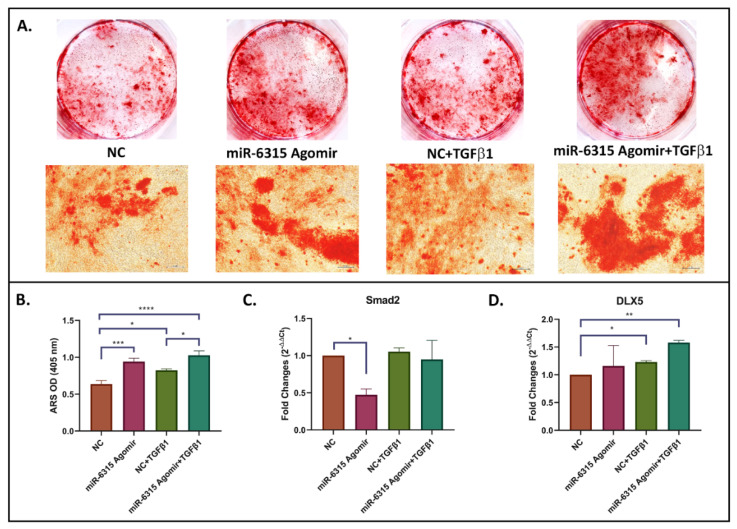
miR-6315 positively modulates osteogenesis partially through influencing TGF-β/Smad2 Signalling. MC3T3.E1 cells treated with NC or miR-6315 agomir were supplemented with/without TGF-β1 during osteoblast differentiation. At Day 21, cells were harvested for the mineralization assay and RT-qPCR analyses. (**A**) Alizarin Red S (ARS) staining. Scale bar on panel A = 200 µm. (**B**) ARS quantification. (**C**) RT-qPCR analyses of Smad2 expression. (**D**) RT-qPCR analyses of distal-less homeobox 5 (DLX5) expression. CycA was used as an internal housekeeping gene. Fold changes of negative control were converted to 1. Statistical significance was marked with * *p* < 0.05, ** *p* < 0.01, *** *p* < 0.001, or **** *p* < 0.0005. Experiments were performed at least three times and results were displayed as the mean ± SEM (*n* = 3). Statistical significance was performed via one-way ANOVA followed by Tukey’s post-test.

In order to verify if miR-6315 influences osteogenesis through TGF-β signalling, MC3T3.E1 cells treated with NC or miR-6315 agomir were supplemented with/without TGF-β1 protein during osteoblast differentiation. At Day 21, cells were harvested for mineralization assay and RT-qPCR analyses. The osteogenic differentiation potential and matrix mineralization were enhanced by miR-6315 treatment (*p* < 0.001, NC vs. miR-6315, Figure 6A,B). When cells were supplemented with TGF-β1 (1 ng/mL), the enhanced osteogenesis and mineralization by miR-6315 treatment was further promoted by TGF-β1 (*p* < 0.0005, NC+ TGF-β1 vs. miR-6315+ TGF-β1, Figure 6A,B).

Additionally, the expression of Smad2 and DLX5 was determined by RT-qPCR at Day 21 of osteoblast differentiation. As shown in Figure 6C, mRNA expression of Smad2 was significantly decreased by miR-6315 treatment (*p* < 0.05, NC vs. miR-6315). The decreased Smad2 mRNA expression caused by miR-6315 treatment was mitigated when cells were co-treated with TGF-β1. However, when cells were supplemented with TGF-β1, no significant difference was found in Smad2 mRNA expression following miR-6315 treatment when compared to NC. On the other hand, no significant difference was found in DLX5 mRNA expression following miR-6315 treatment alone when compared to NC treatment. Furthermore, we noticed a significantly higher DLX5 mRNA expression in NC+TGF-β1 group when compared to the NC group (*p* < 0.05). Consequently, a further increase in DLX5 expression was observed in the miR-6315+TGF-β1 group when compared to the NC group (*p* < 0.01, NC vs. miR-6315+TGF-β1). Collectively, the results indicated that miR-6315 might be able to positively modulate osteogenesis partially through influencing TGF-β/Smad2 signalling.

### 3.7. miR-6315 Regulates Adipogenesis Partially through Modulating TGF-β/Smad2 Signalling

**Figure 7:**
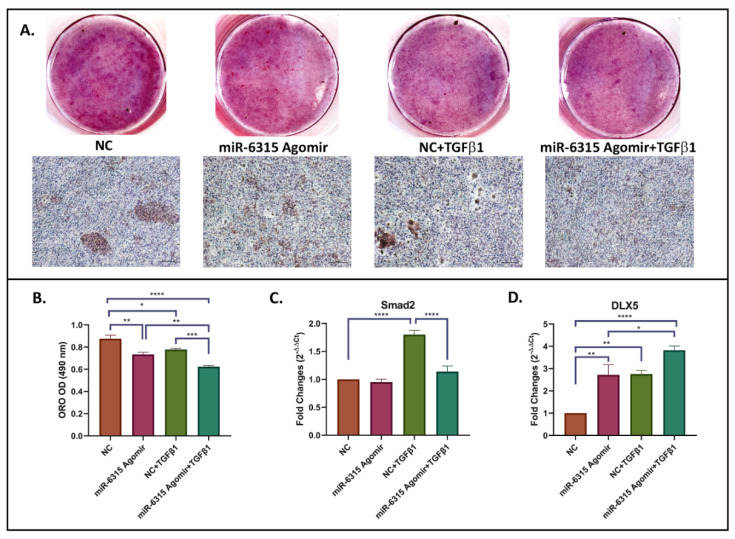
miR-6315 negatively modulates adipogenesis partially through influencing TGF-β/Smad2 signalling. 3T3 F442A preadipocytic cells were treated with NC or miR-6315 agomir and supplemented with/without TGF-β1 during adipocyte differentiation. At Day 14, cells were harvested for adipogenesis assay and RT-qPCR analyses. (**A**) Oil Red O (ORO) staining. Scale bar on panel A = 200 µm. (**B**) ORO quantification. (**C**) RT-qPCR analyses of Smad2 expression. (**D**) RT-qPCR analyses of distal-less homeobox 5 (DLX5) expression. CycA was used as an internal housekeeping gene. Fold changes of negative control were converted to 1. Statistical significance was marked with * *p* < 0.05, ** *p* < 0.01, *** *p* < 0.001, or **** *p* < 0.0005. Experiments were performed at least three times and the results are displayed as the mean ± SEM (*n* = 3). Statistical significance was performed via one-way ANOVA followed by Tukey’s post-test.

To verify whether miR-6315 regulates adipogenesis via influencing TGF-β signalling, 3T3 F442A preadipocytic cells were treated with NC or miR-6315 agomir and were supplemented with/without TGF-β1 during adipocyte differentiation. At Day 14, cells were harvested for adipogenesis assay and RT-qPCR analyses. A significant reduction in lipid droplet formation was observed in the miR-6315-treated group when compared to the NC group (*p* < 0.01, Figure 7A,B). Similarly, when compared to the NC+TGF-β1 treatment group, cells co-treated with miR-6315+TGF-β1 exhibited a notable decrease in lipid accumulation (*p* < 0.001, Figure 7A,B). Additionally, TGF-β1 supplementation has shown significant negative impacts on fat droplet accumulation (*p* < 0.05, NC vs. TGF-β1; *p* < 0.01, miR-6315 vs. miR-6315+TGF-β1). The inhibitory effect on adipogenesis caused by miR-6315 or TGF-β1 alone was further enhanced when cells were co-treated with miR-6315+TGF-β1 (*p* < 0.0005, NC vs. miR-6315+TGF-β1).

Moreover, the relative mRNA expression of Smad2 and DLX5 was measured by RT-qPCR at Day 14 of adipocytic differentiation. While the expression level of Smad2 was not affected by miR-6315 treatment (*p* > 0.05, NC vs. miR-6315, Figure 7C), supplementation of TGF-β1 significantly induced Smad2 expression (*p* < 0.0005, NC vs. NC+TGF-β1). This promotion was attenuated by miR-6315+TGF-β1 co-treatment (*p* = 0.9555, NC vs. miR-6315+TGF-β1; *p* < 0.0005, NC+TGF-β1 vs. miR-6315+TGF-β1). An increase in DLX5 mRNA expression was observed following miR-6315 treatment (*p* < 0.01, NC vs. miR-6315). Cells supplemented with TGF-β1 demonstrated a remarkable increase in DLX5 mRNA expression when compared to the NC treatment group (*p* < 0.01, NC vs. NC+TGF-β1; *p* < 0.0005, NC vs. miR-6315+TGF-β1). These findings implied that miR-6315 might negatively regulate adipogenesis partially through modulating TGF-β/Smad2 signalling.

## 4. Discussion

MTX treatment has often been shown to cause chronic bone-related complications. In both patients and animal models, reduced bone formation and increased marrow adiposity have been observed, and yet the underlying mechanisms are unclear [3,4,13,14]. Previously, using a rat MTX intense treatment model (five daily injections, mimicking intensive MTX treatment for childhood leukemia), our investigations have identified five upregulated miRNA candidates, namely, miR-155-5p, miR-154-5p, miR-344g, miR-6215, and miR-6315, as promising candidates associated with MTX-induced bone defects [24]. Based on bioinformatic analyses, among them, miR-6315 was indicated as a potentially important factor involved in bone/bone marrow fat formation defects following MTX treatment. In this study, we further explored and elucidated the effect and action mechanisms of miR-6315 in regulating osteogenesis/adipogenesis upon MTX treatment. Our MTT and apoptosis assays confirmed that MTX treatment caused significant apoptosis among MC3T3.E1 preosteoblastic cells, which is consistent with the significant apoptotic effect of MTX among osteoblasts, as shown previously in vivo [32]. Additionally, the current study demonstrated that miR-6315 had no significant influence on cell viability and apoptosis among the cultured preosteoblastic cells but exerted a considerable adverse impact on 3T3 F442A preadipocytic cell viability and apoptosis. Furthermore, using in vitro models, osteoblast differentiation and matrix mineralization were enhanced by miR-6315 in preosteoblastic cells, whereas adipogenesis in preadipogenic cells was attenuated by miR-6315 following MTX treatment. Moreover, our results indicate that miR-6315 regulation of osteogenesis/adipogenesis following MTX treatment might be associated with modulation of TGF-β signalling.

### 4.1. miR-6315 Has No Significant Influence on MC3T3.E1 Preosteoblastic Cell Viability and Apoptosis but Significantly Affects 3T3 F442A Preadipocytic Cell Viability and Apoptosis

The numbers and activities of osteoblasts determine the level of bone formation and bone mass. Using a rat short-term MTX administration model, Xian et al. found reduced osteoblast density and proliferation in vivo [32]. MTX at 10 μM has been previously used in in vitro studies, and it was based on the plasma concentration attained in patients under intensive MTX chemotherapy [29]. In the current study, consistently, MTX treatment at 10 μM (MTX+NC) caused a dramatic reduction in preosteoblast viability and an increase in apoptosis when compared to the NC control group. A study conducted by Fan et al. revealed that chronic high-dose MTX treatment was able to decrease osteoblast density in rats, which was found associated with the induction of osteoblast apoptosis rather than a reduction in proliferation [33]. These observations might be due to differences in the cell populations/types seen in the different studies (between preosteoblastic cells here and mature osteoblasts in the Fan et al. study). Previously, increased adipocyte density occurred during the early phase of intensive MTX treatment in rats [33]. Interestingly, a remarkably declined preadipocyte viability and increased apoptosis were observed in the current study with MTX treatment (MTX+NC). These apparently conflicting results may be due to the differences in adipocyte differentiation stages or cell types/populations seen in the two studies (cultured preadipocytes in the current study and adipocytes in the bone marrow in the Fan et al. study).

Some miRNAs have been noted to be closely related to cell proliferation and apoptosis and thus are considered as new targets/tools for the diagnosis, treatment and evaluation of cancers and diseases [34,35,36]. A recent study has shown that miRNA-200b inhibited the proliferation and promoted the apoptosis of cervical cancer cells by directly targeting the Ras homolog family member A (RhoA) gene [37]. miR-485 was found to suppress proliferation and to increase apoptosis of airway smooth muscle cells through modulating TGF-β/Smad signalling in mice with chronic asthma [38]. In the present study, it was found that, while miR-6315 treatment caused no significant changes in preosteoblast viability and apoptosis, it considerably decreased the viability and promoted apoptosis of preadipocytes, which could possibly be related to its attenuating effect on MTX-induced adipogenesis and oil droplet formation (Section 4.2). The regulatory mechanisms of miRNAs might be different in different types of cells. Wang et al. found that miR-21 increased the cell viability and inhibited apoptosis in A549 non-small cell lung cancer cells by modulating the phosphatidylinositol 3-kinase/protein kinase B (PI3K/Akt) pathway [39]. However, Xu et al. demonstrated that miR-21 is positively associated with TNF-α expression, which enhanced the proliferation capability of HeLa cervical cancer cells but had no effect on their apoptosis [40]. The current study revealed that miR-6315 exhibits a greater impact on viability and apoptosis of preadipocytes than preosteoblasts, suggesting that the miR-6315 regulatory mechanisms might be different between preadipocytic cells and preosteoblastic cells.

### 4.2. miR-6315 Alleviates MTX-Induced Reduced Osteogenesis/Increased Adipogenesis

Using a rat acute MTX treatment model, previous studies have shown that administration of MTX caused a reduced trabecular bone volume and an increased marrow adiposity [30,31,32]. Our recent investigation also confirmed the MTX-induced reduced osteogenesis/increased adipogenesis in vitro [24]. Consistent with these previous observations, using MC3T3.E1 preosteoblastic cells and 3T3 F442A preadipocytic cells, the current study also observed the reduced osteogenic differentiation and increased adipogenic differentiation following MTX treatment from the precursor cells.

Recently, mounting evidence has shown that some miRNAs have impacts on bone or marrow fat formation by targeting osteogenesis/adipogenesis-related genes [3]. miR-218 has been characterised as a potent activator of osteogenic lineage commitment and progression by targeting wingless (Wnt) antagonists, including sclerostin, dickkopf-2 (DKK2) and frizzled-related protein 2 (sFRP-2) [41]. Overexpression of miR-218 has been shown to significantly induce the osteoblastogenesis-related gene markers and meanwhile activate transcriptional mediators of Wnt signalling, suggesting that miR-218 positively regulates bone formation through the Wnt signalling pathway [41]. Wang et al. found that elevated levels of miR-214 expression negatively correlated with bone formation and a subsequent study revealed that the role of miR-214 in inhibiting bone formation was via directly binding to activating transcription factor 4 (ATF4) [42]. Similarly, miR-9-5p was observed to be upregulated in bone of osteoporotic patients [43], and overexpressed miR-9-5p was found to decrease osteogenesis and increase adipogenesis by targeting Wnt3a, a ligand that activates Wnt/β-catenin signalling [43].

The current study has firstly addressed the potential roles of miR-6315 in osteogenesis/adipogenesis by using MC3T3.E1 preosteoblastic and 3T3 F442A preadipocytic cells that were treated with/without MTX and then were transfected with miR-6315 or a negative control. By assessing the treatment effects on osteogenesis markers and formation of calcified nodules, our results suggested that miR-6315 promotes osteoblast differentiation and matrix mineralization. Furthermore, miR-6315 along with MTX treatment attenuated the MTX-induced osteogenesis defects. Consistent with our results, some other miRNAs, such as miR-542-3p, miR-217 and miR-10b, have also been shown to positively regulate osteoblast differentiation and matrix mineralization [35,44,45].

Additionally, by measuring the treatment effects on the adipocyte differentiation potentials in 3T3 F442A preadipocytic cells, the remarkable suppression of MTX-induced increased expression of master adipogenic transcription factors C/EBPα and PPARγ and oil droplet accumulation in the MTX+miR-6315-treated group (compared with MTX+NC treated group) indicated the inhibitory effect of miR-6315 in MTX-induced adipogenesis. Thus, our current study suggests that miR-6315 plays a vital role in regulating osteogenesis and adipogenesis and that supplementary treatment with miR-6315 preserves osteoblast differentiation but attenuates adipocyte differentiation in vitro after MTX treatment. Based on these, it could be proposed that the induction of miR-6315 expression in bone of MTX-treated rats, as shown in our recent study, may be associated with its inhibitory effect, attempting to limit the extent of MTX-induced increased adipogenesis, and/or with its osteogenesis-promoting effect, attempting to enhance the bone recovery mechanism following MTX treatment [13,24,32,33].

### 4.3. miR-6315 Regulates Osteogenesis/Adipogenesis at Least Partially via Alternations in TGF-β Signalling

The activation of TGF-β/Smad signalling is highly dependent on the presence of TGF-β ligands. Secreted TGF-β1 affects osteoblast differentiation according to cell incubation conditions, such as the concentration of TGF-β1 and cell density [45]. It was reported that TGF-β1 promotes osteogenic differentiation at low concentrations (0.1–1 ng/mL) but exhibits contrary impacts at high concentrations (e.g., 10 ng/mL) [45]. While TGF-β/Smad signalling has been revealed as a double-edged sword in bone formation [46,47,48], it has been shown to have negative impacts on adipogenesis and fat accumulation [46,49].

Previous work found that miR-6315 might be involved in MTX-induced bone defects by targeting Smad2 [24]. Smad2 is known to respond to TGF-β ligands interacting with the receptor complex, mediating the gene expression of various transcription factors, including osteogenic gene DLX5 (an activator of Runx2) [46]. The current study, through examining the miR-6315 treatment effects on the mRNA and protein expression of Smad2 and DLX5, observed that miR-6315 transfection considerably inhibited Smad2 and DLX5 expression at both transcriptional and post-transcriptional levels in osteoblasts, which indicated downregulation of TGF-β/Smad2 signalling.

As steps to further understand the underlying mechanisms by which miR-6315 regulates bone/fat formation, in the current study, we have investigated the effects of miR-6315 transfection on activation TGF-β signalling pathways in osteoblastic and adipogenic cells. Consistent with previous findings, our results illustrated that osteogenic differentiation potential and matrix mineralization enhanced by miR-6315 were further promoted by 1 ng/mL TGF-β1 supplementation [45]. In agreement with previous work, adipogenesis and oil droplet accumulation suppressed by miR-6315 were shown to be further attenuated with the combination treatment with 1 ng/mL TGF-β1 [45,50,51]. Collectively, our findings suggest that miR-6315 regulates osteogenesis/adipogenesis potentially via modulating TGF-β signalling.

Furthermore, the current study noticed that miR-6315 treatment inhibited the mRNA expression of Smad2 but had no significant effect on expression of downstream transcription factor DLX5 of TGF-β/Smad signalling. However, DLX5 mRNA expression was found to share a similar pattern of changes in Smad2, both with a further increase upon 1 ng/mL TGF-β1 supplementation. These findings may imply that miR-6315 signalling might be associated with multiple factors and/or signalling pathways, which together contribute to the outcomes observed. Recently, several hub genes of miR-6315 were identified, such as S-phase kinase-associated protein 1 (Skp1), activin A receptor type 1 (Acvr1) and protein phosphatase 2 scaffold subunit Aβ (Ppp2r1β) [24]. These factors might not only be linked to each other to form a complex regulatory network but also establish signalling crosstalk between TGF-β signalling and other signalling pathways. For instance, BMP signalling and Wnt signalling have been well characterized to play roles in bone and marrow fat formation, and they have been shown to have interactions with TGF-β signalling [49,50,52,53]. Further studies are needed to understand the roles and action mechanisms of miR-6315 in controlling the balance in osteogenesis/adipogenesis and in the bone loss/marrow adiposity defects caused by cancer chemotherapy. For examples, the use of specific inhibitor(s) for TGF-β receptor-2 (TBRII) and/or receptor-1 (TBRI or ALK5) together with miR-6315 interventions will provide a better understanding of the miR-6315 pathway mechanisms and whether it indeed modulates TGF-β signalling in controlling osteogenesis/adipogenesis and in the bone loss/marrow adiposity defects caused by MTX chemotherapy.

In addition, while the in vivo miRNA-based interventions have not been within the scope of the current study and observations from in vitro experiments may not completely replicate the conditions of cells in the organism, future work is required to conduct the miR-6315-based in vivo validation experiments with MTX-treatment and/or cancer-bearing animal models.

## 5. Conclusions

**Figure 8:**
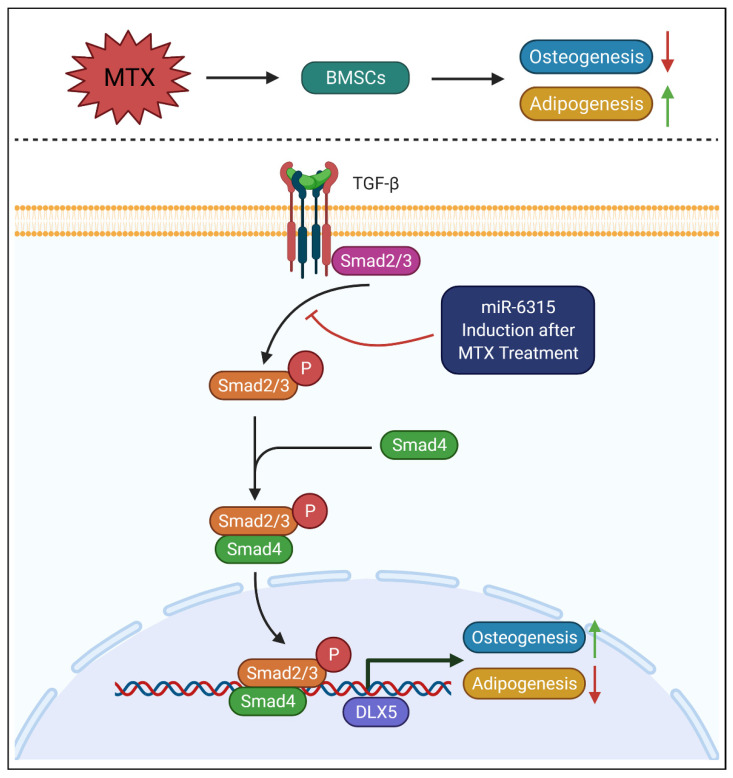
A schematic diagram illustrating the potential role of miR-6315 in regulating osteogenesis and adipogenesis partially through modulating TGF-β/Smad2 signalling. Methotrexate (MTX) treatment leads to upregulated expression of miR-6315 and bone/fat formation switch in bone marrow stromal cells (BMSCs). Activation of miR-6315 in osteoblasts and adipocytes after MTX treatment results in the inhibition of Smad2 and alternation in TGF-β/Smad2 signalling, which were found to be associated with the effect of miR-6315 in attenuating MTX treatment-induced reduced osteogenesis and increased adipogenesis.

Taken together, this study has shown that the MTX chemotherapy-induced bone/fat switch in the bone marrow may be associated with expression and function of miR-6315 in the bone (Figure 8). Our findings indicate that miR-6315 treatment might attenuate MTX-induced decreased osteogenesis and increased adipogenesis via targeting Smad2, which may lead to partial suppression of TGF-β signalling. This study has demonstrated a potential role of miR-6315 in ameliorating MTX treatment-induced reduced osteogenesis and increased adipogenesis and thus has shed light on the molecular mechanisms of MTX therapy-associated bone/fat switch. From the results of our study, it can be proposed that the induction of miR-6315 expression in bone might be correlated with the potential process of limiting MTX treatment-induced adipogenesis and promoting osteogenesis for bone recovery following MTX treatment. However, future in vivo studies will be required to address whether supplementary treatment of miR-6315 can be a therapeutic approach to prevent/attenuate MTX-associated bone loss and marrow adiposity.

## Figures and Tables

**Table 1:** Primer sequences used for RT-qPCR.

Gene	Forward Primer (5′-3′)	Reverse Primer (5′-3′)
RUNX2	CCCAGCCACCTTTACCTACA	TATGGAGTGCTGCTGGTCTG
ALP	GCTGATCATTCCCACGTTTT	CTGGGCCTGGTAGTTGTTGT
OSX	ACTCATCCCTATGGCTCGTG	GGTAGGGAGCTGGGTTAAGG
OCN	AAGCAGGAGGGCAATAAGGT	TTTGTAGGCGGTCTTCAAGC
C/EBPα	TGGACAAGAACAGCAACGAG	CCTTGACCAAGGAGCTCTCA
PPARγ	TTTTCAAGGGTGCCAGTTTC	AATCCTTGGCCCTCTGAGAT
Smad2	GGAACCTGCATTCTGGTGTT	ACGTTGGAGAGCAAGCCTAA
DLX5	CCACCAGCCAGCCAGAGAAA	GGGGCATCTCCCCGTTTTT
CycA	CGTTGGATGGCAAGCATGTG	TGCTGGTCTTGCCATTCCTG

RUNX2: runt-related transcription factor 2; ALP: alkaline phosphatase; OSX: osterix; OCN: osteocalcin; C/EBPα: CCAAT/enhancer binding protein alpha; PPARγ: peroxisome proliferator activated receptor gamma; Smad2: SMAD family member 2; DLX5: distal-less homeobox 5; CycA: cyclophilin A.

## Data Availability

The data that support the findings of this study are available on request from the corresponding author.
